# Utilizing the Promise of Omadacycline in a Resistant, Non-tubercular Mycobacterial Pulmonary Infection

**DOI:** 10.7759/cureus.5112

**Published:** 2019-07-09

**Authors:** Ramandeep Minhas, Shaurya Sharma, Suhali Kundu

**Affiliations:** 1 Diagnostic Radiology, SUNY Downstate Medical Center, Brooklyn, USA; 2 Internal Medicine, Maimonides Medical Center, Brooklyn, USA

**Keywords:** mycobacterium, pulmonology, omadacycline, non-tubercular, resistant, antibiotics and antibiotic resistance, mycobacterium abscessus, novel antibacterial

## Abstract

The non-tubercular mycobacterium, Mycobacterium abscessus (M. abscessus), is a resistant, opportunistic pathogen that causes lung infections that are not curable, but which can be controlled with appropriate antibiotic regimens. A new broad-spectrum tetracycline analog called omadacycline was approved in 2018 in the United States for the treatment of bacterial, community-acquired pneumonia and acute skin infections. We discuss a case to assess the effect of this novel antibiotic in the management of a nefariously resistant M. abscessus infection. Our patient had underlying chronic bronchiectasis and a long-standing M.abscessus infection, along with numerous drug allergies and previously failed antibiotic regimens. All of these factors entailed a disease process that was difficult to manage and a worsening morbidity. The utilization of omadacycline in this case addressed a multitude of problems by improving ease of administration and circumventing the patient’s allergic reaction to antibiotics; on follow-up, the patient demonstrated an improved clinical status including well-controlled symptoms and weight gain.

## Introduction

The non-tubercular mycobacterium, M. abscessus, is a resistant, opportunistic pathogen that causes pulmonary infections that can only be managed, rather than cured. It usually affects patients with an underlying chronic disease process such as bronchiectasis or cystic fibrosis. A new tetracycline analog called omadacycline, recently approved for bacterial skin/soft-tissue infections and community-acquired pneumonia, has also shown promise in the management of nefariously resistant non-tubercular mycobacterial infections. We present and discuss a case wherein omadacycline was utilized in a patient with a long-standing Mycobacterium abscessus infection.

## Case presentation

A 67-year-old Cantonese-speaking woman from China (Shenzhen region) with a medical history of hypertension and chronic bronchiectasis presented with a two-week history of worsening left-ear pain in addition to chronic shortness of breath and cough productive of yellow-tinged sputum.

The patient denied fever, chills, or headaches. On further questioning, she admitted having night sweats, and a decreased appetite accompanied by a 10-pound weight loss over three months.

Notably, she had documented allergies to Imipenem, cefoxitin, quinolones, tigecycline, yobramycin, and doxycycline.

**Figure 1:**
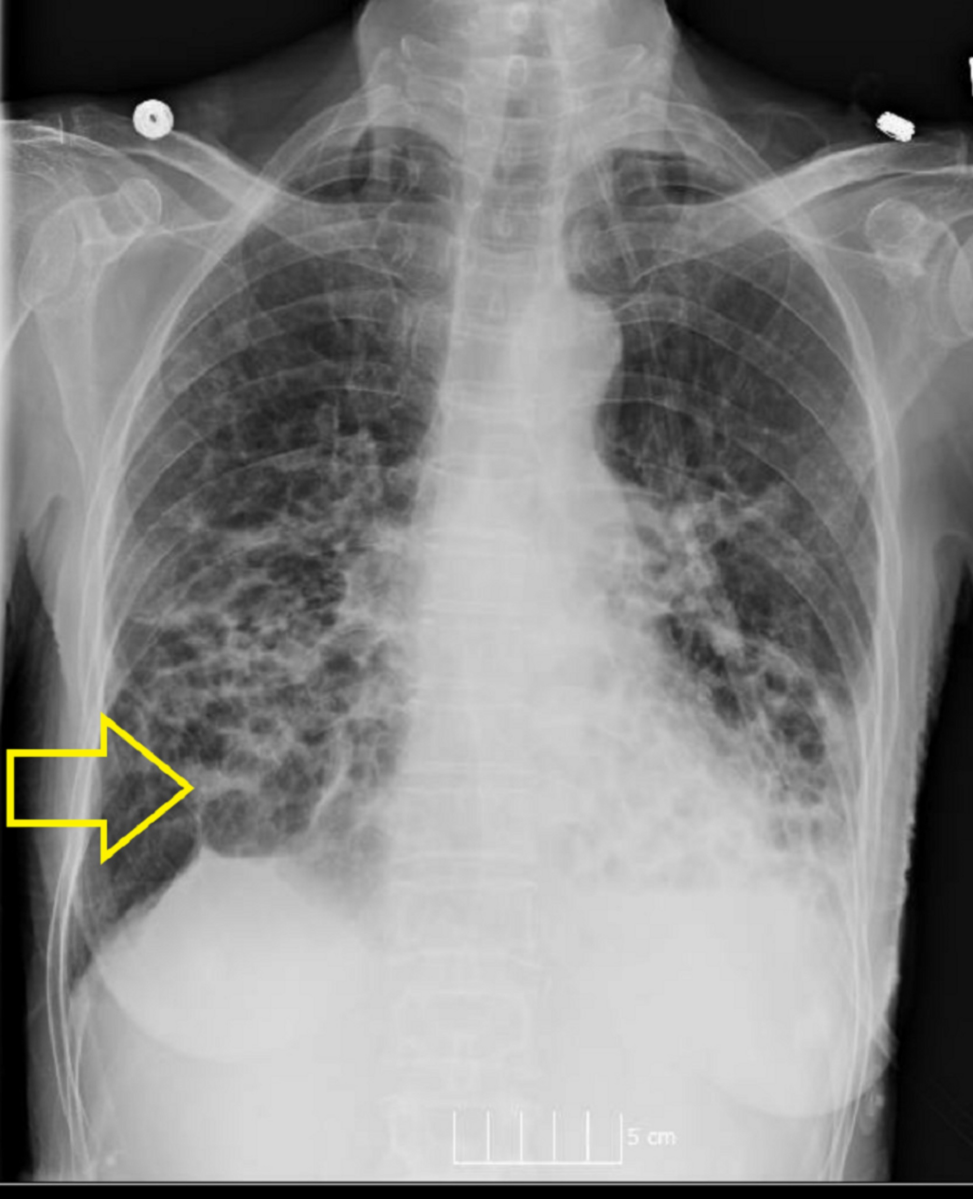
Chest radiography showing extensive cystic changes and bronchiectasis.

**Figure 2:**
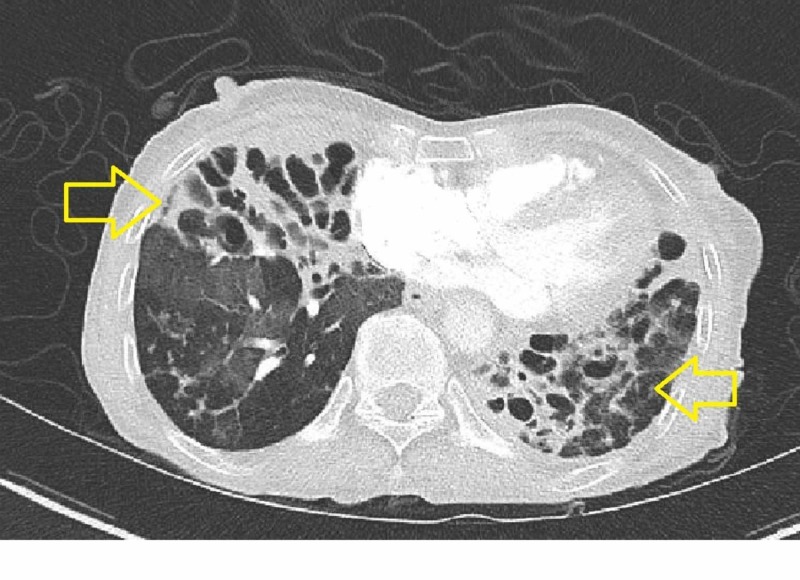
Computed tomography chest with IV contrast (axial) demonstrating bilateral cystic bronchiectasis with fibrosis indicative of chronic, advanced M. abscessus infection.

She had emigrated from China in 2003 and had failed prior treatment for chronic bronchiectasis with M.abscessus with aztreonam alone as well as combination therapy with aztreonam and doxycycline. Chest imaging (radiography and tomography) showed extensive pulmonary disease (Figures 1-2).

**Figure 3:**
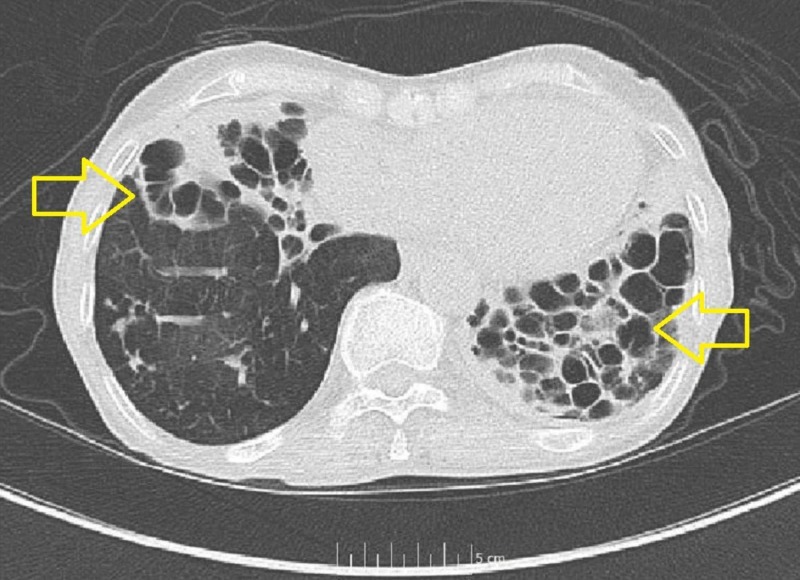
Repeat CT chest without contrast, read as extensive long-standing cystic bronchiectasis and fibrosis with overall appearance stable and unchanged from previous imaging.

Given that the patient has failed treatment earlier and her extensive medication allergies, infectious disease (ID) service was consulted for recommendations on alternative therapies. She was started on omadacycline 150 mg per oral route daily in addition to amikacin 500 mg IV three times a week, and aztreonam 1g IV q8 hourly to complete a four-week course. The patient tolerated the therapy well and, upon follow-up, was noted to have improved dyspnea on exertion and a five-pound weight gain. The patient was followed up after a month in our ID clinic and was documented as being clinically stable with much improved cough. Liver-function tests were within normal range and a repeat CT chest without contrast showed stable disease with no progression (Figure 3).

## Discussion

M. abscessus is an opportunistic pathogen that is pervasive in the environment, primarily causing infections in humans with compromised lung function. M. abscessus is the second-most-common non-tubercular mycobacterial infection after the Mycobacterium avium complex [1]. M. abscessus is infamous in the medical literature for being a highly resistant pathogen. This is due to several factors: firstly, a dynamic, open pan-genome that allows the bacteria to evolve and adapt to stressful environmental conditions; secondly, intrinsic resistance mechanisms; and lastly, an ability to survive both intra-cellulary and extra-cellulary within macrophages and caseous lesions, respectively. Therefore, M. abscessus is resistant to a majority of beta-lactams, tetracyclines, aminoglycosides, and macrolides. Anti-tubercular drugs such as isoniazid and rifampicin have been shown to be ineffective as well [1, 2].

Current treatment guidelines recommend a macrolide antibiotic combined with amikacin with cefoxitin, imipenem, or tigecycline. However, recurrence rates of infections with M. abscessus longer than 12 months with the current regimen now stand at 50 percent [3, 4]. Macrolides are known to be the most successful antibiotics in treating infections caused by non-tubercular mycobacteria and are widely prescribed in multi-drug regimens [1]. However, these antibiotics have been unsuccessful in the treatment of infections caused by M.abscessus due to its inducible macrolide resistance, which is conferred by the ribosomal methyl transferase gene erm (41) as well as acquired macrolide resistance due to mutations in the 23S rRNA gene (rrl) [3]. Poor treatment outcomes are attributable to the resistance mechanisms utilized by the bacteria to render the medications ineffective.

Tetracyclines are another class of antibiotics often used in multi-drug regimens for M. abscessus. Among them, omadacycline, a novel tetracycline derivative approved by the FDA in 2018, demonstrated similar in-vitro activity against the M. abscessus complex as tigecycline [5]. It is an aminomethyl tetracycline that can be administered intravenously or orally and achieves excellent concentrations in pulmonary tissues.

In a comparison study between omadacycline and tigecycline, omadacycline was found to have higher and better sustained concentrations in plasma, epithelial lining fluid, and alveolar cells. The minimum inhibitory concentration (MIC) of omadacycline was less than or equal to that of doxycycline and amikacin when compared with various M. abscessus complex isolates. Thus, omadacycline is a promising antibiotic for the treatment of lower respiratory-tract bacterial infections caused by resistant M.abscessus [6, 7]. It incapacitates the common efflux and ribosomal mechanisms of resistance in tetracyclines and has an extended spectrum of activity [8]. Further, the availability of omadacycline in oral formulations and its favorable side-effect profile make it advantageous over tigecycline. The side-effect profile of omadacycline includes nausea and vomiting, hypersensitivity/photosensitivity, and deranged liver-function tests. Omadacycline does not show cross-resistance with beta-lactams, aminoglycosides, polymyxins, or fluoroquinolones [9]. These characteristics of Omadacycline make it a promising alternative in the management of non-tubercular mycobacterial infections amidst increasing antibiotic resistance.

## Conclusions

Omadacycline can be utilized as part of a multi-drug regimen for mitigating and treatment of non-tubercular mycobacterial infections that are increasing in incidence, prevalence, and resistance worldwide.
